# The intrathecal CD163-haptoglobin–hemoglobin scavenging system in subarachnoid hemorrhage

**DOI:** 10.1111/j.1471-4159.2012.07716.x

**Published:** 2012-06

**Authors:** James Galea, Garth Cruickshank, Jessica L Teeling, Delphine Boche, Patrick Garland, V Hugh Perry, Ian Galea

**Affiliations:** * Brain Injury Research Group, Manchester Academic Health Sciences Centre, Salford Royal NHS Foundation TrustManchester, UK; † University of Birmingham & University Hospital BirminghamEdgbaston, Birmingham, UK; ‡ Centre for Biological Sciences, Faculty of Natural and Environmental Sciences, University of SouthamptonSouthampton, UK; § Clinical Neurosciences, Clinical and Experimental Sciences, Faculty of Medicine, University of SouthamptonSouthampton, UK; ¶ Wessex Neurological Centre, Southampton General HospitalSouthampton, UK

**Keywords:** CD163, haptoglobin, hemoglobin scavenging, perivascular macrophage, subarachnoid hemorrhage, vasospasm

## Abstract

Delayed cerebral ischemia resulting from extracellular hemoglobin is an important determinant of outcome in subarachnoid hemorrhage. Hemoglobin is scavenged by the CD163-haptoglobin system in the circulation, but little is known about this scavenging pathway in the human CNS. The components of this system were analyzed in normal cerebrospinal fluid and after subarachnoid hemorrhage. The intrathecal presence of the CD163-haptoglobin–hemoglobin scavenging system was unequivocally demonstrated. The resting capacity of the CD163-haptoglobin–hemoglobin system in the normal CNS was 50 000-fold lower than that of the circulation. After subarachnoid hemorrhage, the intrathecal CD163-haptoglobin–hemoglobin system was saturated, as shown by the presence of extracellular hemoglobin despite detectable haptoglobin. Hemoglobin efflux from the CNS was evident, enabling rescue hemoglobin scavenging by the systemic circulation. Therefore, the CNS is not capable of dealing with significant intrathecal hemolysis. Potential therapeutic options to prevent delayed cerebral ischemia ought to concentrate on augmenting the capacity of the intrathecal CD163-haptoglobin–hemoglobin scavenging system and strategies to encourage hemoglobin efflux from the brain.

Subarachnoid hemorrhage (SAH) is a life-threatening condition, and causes significant neurological sequelae in survivors. Delayed cerebral ischemia (DCI) occurs in up to 30% of patients surviving the initial hemorrhage and is a leading cause of morbidity and mortality (Vergouwen *et al.* 2010). There is strong evidence and universal consensus that the principal determinant of DCI is extracellular hemoglobin (Hb) released from extravasated erythrocytes (Pluta *et al.* 2009).

The predominant pathway for Hb clearance is the CD163-haptoglobin–hemoglobin (CD163-Hp–Hb) system. In the circulation, any extracellular Hb is immediately bound by haptoglobin (Hp) with extremely high affinity (*K*_d_ of ∼1 pM) (Hwang and Greer 1980). Hp–Hb complexes are recognized by CD163 expressed on tissue macrophages and circulating monocytes, resulting in receptor-mediated endocytosis (Kristiansen *et al.* 2001). Despite the presence of lower affinity Hb-scavenging systems such as cubilin and megalin (Gburek *et al.* 2002) and downstream heme metabolism via hemopexin-CD91 (Hvidberg *et al.* 2005), the CD163-Hp–Hb system plays a critical non-redundant role (Maniecki *et al.* 2008).

The basic building block of Hp consists of an α and a β chain. Two Hp alleles exist (Hp1 and Hp2) which differ by the number of free cysteine residues on the α chain (1 or 2 residues) capable of forming intermolecular disulphide binds. Therefore, individuals homozygous for the Hp1 allele form Hp dimers (a phenotype denoted in this study as Hp1-1) while individuals heterozygous or homozygous for Hp2 synthesize Hp trimers, tetramers and/or higher order multimers (phenotypes denoted in this study as Hp2). Hp is not synthesized in the normal CNS (Kumar *et al.* 2010) but is detectable within the CSF with a pattern suggestive of leakage across blood–CNS barriers (BCB) (Chamoun *et al.* 2001).

In the rodent CNS, CD163 is exclusively expressed by meningeal, perivascular and choroid plexus macrophages. By virtue of their location in contact with subarachnoid and perivascular blood products, these macrophages are ideally placed to clear extracellular Hb. The components of the CD163-Hp–Hb system are present within the CNS but their dynamics have been poorly studied. Therefore, a detailed study was undertaken in the healthy state and after SAH.

## Materials and methods

### Patients

Patients were recruited after referral to tertiary centres in Manchester, Birmingham, Southampton and Cambridge after approval by the respective Research Ethical Committees. The demographics and clinical characteristics of control individuals (*n* = 20) and patients with SAH (*n* = 30) are listed in Table 1. CSF in patients with SAH was obtained from external ventricular drains. Control individuals were patients with non-inflammatory/non-hemorrhagic conditions who underwent lumbar puncture and were subsequently found to have normal CSF with respect to protein, glucose, cell count, cytology, albumin CSF/serum quotient and isoelectric focusing for oligoclonal bands.

**Table 1:** Demographics

	SAH	Controls	*p*
Number	30	20	
Age in years	52.1 ± 9.6	41.3 ± 16.6	*p* = 0.006^b^
Sex (% female)	68	70	*p* = 1^c^
Hp genotype (% Hp1-1, Hp2-1, Hp2-2)	13, 83, 4	20, 75, 5	*p* = 0.37^c^
Presentation Glascow Coma Score^a^	11 ± 4.2		
Fisher grade^a^	3.7 ± 0.7		
WFNS Score^a^	2.9 ± 1.5		
DCI: clinical evidence^a^	6/26		
DCI: CT evidence^a^	6/26		
DCI: clinical and CT evidence^a^	8/26		
Glasgow Outcome Score	3.5 ± 1.7		
Days post-ictus when sample taken	3.9 ± 2.6		

Values represent mean ± standard deviation where applicable.

a Data only available for 26/30 patients.

b Student’s *t*-test.

c Fisher exact test.

### Immunoassays

Serum and CSF soluble CD163, and CSF Hp levels were analyzed by enzyme-linked immunosorbent assay (ELISA) using commercially available kits, as per kit instructions (Macro163, IQP-383, IQ Products, Netherlands and AssayMax Human Haptoglobulin, EH2003-1, AssayPro, MO, USA). Serum albumin, CSF albumin and serum Hp were analyzed by rate nephelometry on a Beckman Coulter IMMAGE immunochemistry system.

### Hp phenotyping

Hp phenotyping was performed using denaturing discontinuous sodium dodecyl sulphate (SDS) polyacrylamide gel electropheresis followed by western blotting with modifications of a technique described previously (Beutler *et al.* 2002). Briefly, 2 μL of human serum was boiled for 5 min at 95°C in 40 μL of sample loading buffer [1% SDS, 10% glycerol, 25 mM Tris (pH6.8), 0.005% bromphenol blue, 5%β-mercaptoethanol]. Twenty microliters was loaded onto a 15% polyacrylamide gel, and electrophoresis performed at 200 V with Laemmli running buffer (25 mM Tris, 192 mM glycine, 0.1% SDS, pH8.3). Samples were then electroblotted onto a nitrocellulose membrane (Hybond; GE, Little Chalfont, Buckinghamshire, UK) overnight at 30 V in Laemmli buffer with 20% methanol. The membranes were blocked for 1 h with 3% albumin in Tris-buffered saline (20 mM Tris, 150 mM NaCl, pH8) containing 0.5% Tween-20 (TBS-T) and probed for 1 hr with 1 : 5000 polyclonal rabbit anti-Hp antibody (Sigma, Gillingham, Dorset, UK). After 3 washes in TBS-T, the membranes were incubated with 1 : 10 000 polyHRP-conjugated goat anti-rabbit immunoglobulin (Pierce, Thermo Scientific, Cramlington, Northumberland, UK) for 1 h, washed in TBS-T and developed with 0.05% 3,3′-diaminobenzidine, 0.015% hydrogen peroxide and 0.1 M phosphate buffer. All incubations were performed in blocking solution at 20°C. Hp is 16% glycosylated (Mann *et al.* 1994) and the α1, α2 and β chains, which have predicted molecular weights of 40, 16 and 9 kDa, ran at 42, 20 and 16 kDa respectively, as described previously (Berkova *et al.* 2001).

### Derivative spectrophotometry

Sufficient CSF was available from 23 patients with SAH to perform derivative spectrophotometry for quantification of heme products. Scanning spectrophotometry between 350 and 600 nm was performed in 1 nm increments on 125 μL of neat CSF in a 96 well Corning flat-bottomed plate on a Varioskan Flash spectrophotometer (Thermo Scientific, Cramlington, Northumberland, UK) with pathlength correction to 1 cm. An average of 10 readings was taken per sample. Concentrations of oxyhemoglobin, bilirubin and methemoglobin were calculated using iterative modelling software kindly provided by Freek Roelandse at Leiden University Medical Centre (Duiser *et al.* 2001).

### CT volumetric analysis of blood load

Computed tomography (CT) images were available for 20 patients. Quantitative analysis of bleed size was performed using MIPAV (Medical Image Processing, Analysis and Visualization) Version 5.3.1 (http://mipav.cit.nih.gov/). Matched unenhanced CT scan images were analysed sequentially. The entire CT scan sequence was used with images taken at 5-mm intervals in the orbito-meatal plane. Bone and calcified tissues (such as choroid plexus) were cropped from the DICOM images. Assessment of blood load was performed by determining the average voxel radiodensity, its standard deviation and the sum of voxel intensities. For qualitative assessment of SAH, two independent observers graded the extravasated blood volume visually, with one being the lowest blood load and 20 being the highest. The correlation between the measured and the observed blood load was good (correlation coefficient 0.95).

### Delayed cerebral ischemia

Clinical information regarding DCI was available for 25 patients. We defined clinical DCI as the onset of a new focal neurological deficit or a two point drop in the Glascow Coma Score in the absence of rebleeding, hydrocephalus, metabolic abnormalities or seizure activity. CT evidence of DCI consisted of low attenuation on unenhanced CT of the brain consistent with ischemia, irrespective of clinical state, which was not deemed to be a result of surgical intervention.

### Statistical analysis

Graphpad Prism 5 was used for statistical analysis and graph preparation. The distribution of each dataset was tested, and parametric (unpaired Student’s *t*-test) or non-parametric (Mann–Whitney) tests were employed accordingly. For comparison between more than two groups, an extremes-of-outcome approach was used. Cases were divided into tertiles, and the null hypothesis was applied to the highest and lowest tertiles using the appropriate parametric or non-parametric test. The null hypothesis was rejected at *p* < 0.05.

## Results

### The CNS has a poor total Hp Hb-binding capacity

Hp was readily detectable in CSF of control patients, with a mean concentration of 834 ng/mL (range 69–2081 ng/mL). We developed the concept of total Hb-binding capacity (THBC) of Hp as a useful measure of the immediate reserve available to deal with extracellular Hb; it is expressed as Hb mass. Based on a CSF volume of 150 mL and an average CSF Hp concentration of 834 ng/mL, the normal CNS has a THBC of ∼100 μg Hb (see Appendix S1 for calculation). In contrast, the circulation has a THBC of ∼5 g Hb (based on a blood volume of 5 L and an average serum Hp concentration of 1.36 g/L). Therefore, the CNS has a poor THBC, approximately 50 000-fold lower than that of the circulation.

### The CNS total Hp Hb-binding capacity is not dependent on Hp phenotype

Hp2-containing species (Hp2-1 trimers and Hp2-2 multimers) have larger Stokes-Adam radii than Hp1-1 (a dimer); this has been shown to influence their penetration into the CSF (Chamoun *et al.* 2001). However, Hp mass concentration, not molar concentration, reflects the capacity of Hb binding. When expressed in ng/mL, there was no significant difference in CSF Hp concentration between Hp1-1 and Hp2 control patients (mean of 892 ng/mL in Hp1-1 and 819 ng/mL in Hp2, *n* = 4 in Hp1-1 and *n* = 15 in Hp2), showing that THBC in normal CSF is not dependent on Hp phenotype. Lower CSF molar concentrations of Hp2-containing species are thus compensated by higher Hb-binding stoichiometries.

### Intrathecal synthesis of soluble CD163 indicates a substantial population of CD163-positive cells in the normal CNS

In the circulation, sCD163 is constitutively shed from CD163-positive cells (Sulahian *et al.* 2001) and serum sCD163 levels correlate with the number of macrophages present in the body (Moller *et al.* 2002; Maniecki *et al.* 2008). In the absence of inflammation, sCD163 is therefore indicative of the presence and number of CD163-positive cells, and therefore Hb scavenging capacity.

**Fig. 1:**
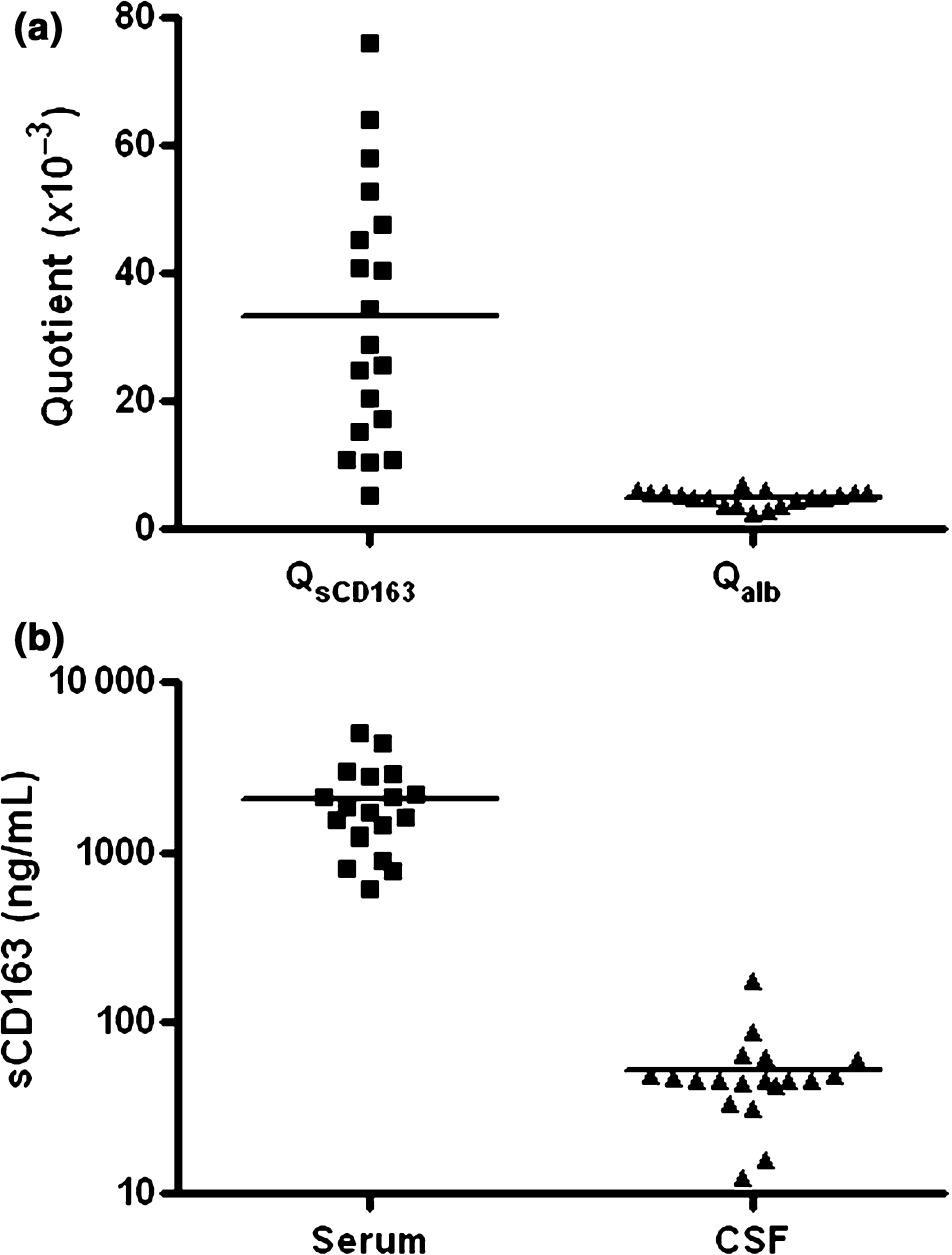
Soluble CD163 in the healthy CNS. (a) sCD163 and albumin CSF/serum quotients (*n* = 19, *p* < 0.0001). (b) CSF and serum sCD163 levels (*n* = 19, *p* < 0.0001).

To obtain a quantitative estimate of CD163-positive cells within the CNS, sCD163 was assayed in CSF of control patients. sCD163 was readily detectable with a mean concentration of 53.2 ng/mL (range 12.6–176.0 ng/mL). To assess the intrathecal synthesis of sCD163, the CSF/serum quotient (see Appendix S1 for calculation) of sCD163 (*Q*_sCD163_) was compared with that of albumin, a molecule of similar size and solubility which is not synthesized within the CNS; this method is typically employed to distinguish intrathecal synthesis from passive diffusion across the BCB (Reiber 2010). Pairwise comparison showed that the *Q*_sCD163_ was on average seven times higher than *Q*_alb_ (Fig. 1a) and calculations showed that ∼77% of sCD163 was produced intrathecally (see Appendix S1 for calculation). This demonstrates that the majority of sCD163 in the CSF was derived from a population of CD163-positive cells within the CNS.

Pairwise comparison showed that CSF sCD163 levels were ∼50 times lower than those in serum in normal individuals (Fig. 1b). Based on CSF and plasma volumes of 155 mL and 5 L, the CNS contains a total of ∼0.08 nmol sCD163 while the circulation carries ∼100 nmol, a 1200-fold difference. This is consistent with the relative paucity of macrophages in the normal CNS compared with the rest of the body.

### Macrophage recruitment to the CNS boosts intrathecal Hp–Hb scavenging

**Fig. 2:**
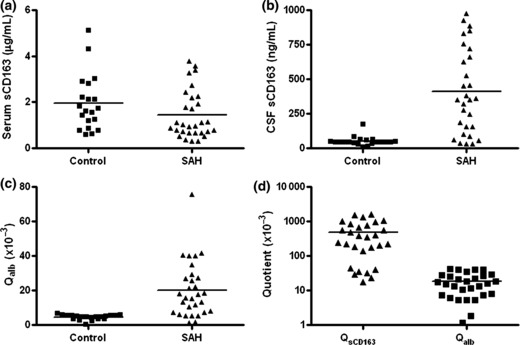
Soluble CD163 in SAH. (a) Serum sCD163 levels in SAH patients (*n* = 30) and controls (*n* = 20) (*p* > 0.05). (b) CSF sCD163 levels in SAH patients (*n* = 29) and controls (*n* = 20) (*p* < 0.0001). (c) BCB compromise in SAH (*n* = 30) as shown by albumin permeability versus controls (*n* = 20) (*p* < 0.001). (d) sCD163 and albumin CSF/serum quotients in SAH (*n* = 29, *p* < 0.0001).

In a variety of inflammatory conditions, serum levels of sCD163 are elevated (Moller *et al.* 2002). As SAH is accompanied by a systemic acute phase response (Jernas *et al.* 2009), it was important to establish whether circulating sCD163 levels were raised in SAH. There was no significant difference in serum sCD163 levels between SAH patients and controls (Fig. 2a). However, sCD163 was significantly increased in CSF from SAH patients, around eight times higher than control CSF (Fig. 2b). The higher CSF sCD163 may have resulted from increased influx from the circulation across a dysfunctional BCB or increased intrathecal shedding. The BCB integrity, as measured by *Q*_alb_, was significantly compromised in SAH compared with control patients (Fig. 2c). However, pairwise comparison showed that the *Q*_sCD163_ was on average 35 times higher than *Q*_alb_ (Fig. 2d) and calculations showed that ∼ 84% of sCD163 was produced intrathecally. This demonstrates that the majority of sCD163 in the CSF of SAH patients was derived from CD163-positive cells within the CNS.

**Fig. 3:**
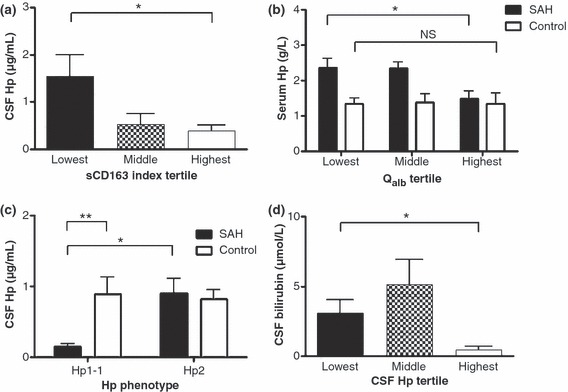
Haptoglobin in SAH. (a) Higher sCD163 index was significantly associated with a lower CSF Hp level (*n* = 9 upper tertile, *n* = 9 lower tertile, *p = 0.02). (b) Lower serum Hp levels with higher albumin quotients in SAH (*n* = 9 upper tertile, *n* = 9 lower tertile, *p = 0.02) but not controls (*n* = 6 upper tertile, *n* = 6 lower tertile, *p* > 0.05). (c) Absolute hypohaptoglobinorrhachia after SAH in the Hp1-1 individuals (*n* = 4) versus Hp2 SAH patients (*n* = 25, *p = 0.04) and Hp1-1 controls (*n* = 4, **p = 0.02). (d) Association between lower CSF Hp and higher CSF bilirubin levels (*n* = 9 upper tertile, *n* = 9 lower tertile, *p = 0.02).

The enhanced intrathecal generation of sCD163 after SAH may potentially result from increased shedding or enhanced influx of monocytes/macrophages into the CNS, or both. It is possible to discern the relative contribution of these two processes because they have divergent effects on scavenging of Hp–Hb complexes. Increased shedding interferes with Hp–Hb scavenging (Schaer *et al.* 2007) while enhanced cellular influx into the CNS would be expected to increase scavenging. A higher sCD163 index was significantly associated with a lower CSF Hp level (Fig. 3a), indicating that recruitment of monocytes/macrophages to the CNS was occurring in SAH with a resultant increase in Hp–Hb scavenging.

### Saturation of the intrathecal CD163-Hb–Hp system in SAH

The mean CSF Hp concentration in SAH cases was 799 ng/mL which on calculation is equivalent to a Hb-binding capacity of 15 × 10^−12^ mol/mL (see Appendix S1 for calculation). Pairwise comparison of the Hb-binding capacity and Hb concentration in SAH cases showed that Hb concentration was on average 150 times higher than the THBC, indicating that the majority of Hb was not bound to Hp. In the circulation, ahaptoglobinemia is a hallmark of severe intravascular hemolysis, and hemoglobinemia only becomes detectable when Hp is completely consumed. However Hp was still detected in the CSF of SAH patients, despite high levels of free Hb. This indicates saturation of the intrathecal CD163-Hb–Hp scavenging system, with a residual inability to deal effectively with free Hb. Clearly, other lower affinity intrathecal Hb-scavenging systems were unable to cope with the amounts of extracellular Hb present in the subarachnoid space.

### Evidence for Hb efflux and peripheral scavenging

In SAH, the concentration gradient for Hb across the BCB is extremely steep because of immediate systemic scavenging. As one of the major determinants of permeability across the BCB is the concentration gradient, conditions are ideal for continuous diffusion of Hb out of the CNS for scavenging by a vastly more capacious systemic THBC. We sought evidence for such a process, which would result in a relative decrease in circulating Hp levels in proportion to BCB permeability. Indeed, SAH patients with a higher CSF/serum albumin quotient had significantly lower serum Hp levels; no such relationship was observed in control patients (Fig. 3b).

### Hypohaptoglobinorrhachia occurs in SAH

Hp is an acute phase reactant. As expected, serum levels of Hp in patients with SAH were significantly elevated (×1.6) compared with control patients (means of 2.1 g/L vs. 1.36 g/L, *p* = 0.0008). CSF Hp levels in SAH were not higher than control patients (means of 799 ng/mL vs. 834 ng/mL). When patients with different Hp phenotypes were analyzed separately, a significantly lower CSF Hp was noted in the Hp1-1 SAH group compared with the Hp1-1 control and Hp2 SAH groups (Fig. 3c). The absolute hypohaptoglobinorrhachia in Hp1-1 SAH patients had a magnitude of ∼×6. CSF Hp did not change in the Hp2 SAH group (Fig. 3c) – the absence of an increase in CSF Hp in the face of increased serum Hp suggests a relative hypohaptoglobinorrhachia in Hp2 SAH patients.

### Mechanism of hypohaptoglobinorrhachia

The marked hypohaptoglobinorrhachia in the Hp1-1 group versus the Hp2 group was not secondary to a larger bleed size in the Hp1-1 group (average voxel radiodensity of 99 and 108 in Hp1-1 and Hp2 groups). Nor could it be explained by a significantly higher CNS macrophage load/activity, as shown by comparable sCD163 indices (38.2 and 33.4 in Hp1-1 and Hp2 groups, see Appendix S1 for calculation). Preferential efflux of Hp1-1-Hb complexes is unlikely because evidence of reduced serum Hp levels in Hp1-1 patients to support this explanation was lacking (2.45 g/L and 2.08 g/L in Hp1-1 and Hp2 groups). In addition, there was no difference in average voxel density, sCD163 index and serum Hp when all patients were analyzed by their CSF Hp concentration (data not shown).

The most likely explanation underlying the marked hypohaptoglobinorrhachia in Hp1-1 SAH patients is enhanced uptake of Hp1-1–Hb complexes by CD163-positive macrophages within the CNS, as has been demonstrated *in vitro* (Asleh *et al.* 2003). To seek evidence for this explanation, CSF bilirubin levels were studied because increased intrathecal internalization of Hp–Hb complexes results in higher CSF bilirubin levels (Clark and Sharp 2006). There was a tendency towards higher CSF bilirubin levels in the Hp1-1 group despite a lower albumin quotient, although this did not reach statistical significance (bilirubin of 3.31 and 3.03 μmol/L in Hp1-1 and Hp2 groups; *Q*_alb_ of 0.017 and 0.021 in Hp1-1 and Hp2 groups). In addition, there was a clear statistically significant association between lower CSF Hp and higher CSF bilirubin levels when all SAH patients were analyzed together, despite a lower albumin quotient (Fig. 3d).

### Hypohaptoglobinorrhachia is associated with protection from DCI

**Fig. 4:**
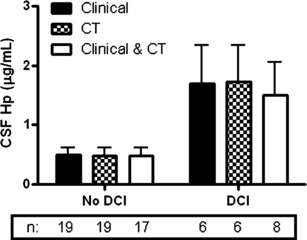
CSF Hp levels and DCI in SAH (*n* for each subgroup indicated below graph). Higher Hp levels in patients with delayed cerebral ischemia.

Clinical details and CSF haptoglobin levels were both available for 25 patients, enabling the occurrence of DCI to be assessed in relation to CSF haptoglobin levels in this subgroup. Consistent with the established protective effect of the Hp1-1 phenotype from vasospasm (Borsody *et al.* 2006), all seven patients who developed clinical and/or radiological features of DCI in the present study had the Hp2 phenotype. We found that mean CSF Hp levels were three times lower in SAH patients without clinical and/or radiological evidence of DCI (Fig. 4). Eighteen patients received interleukin-1 receptor antagonist between CSF sampling and occurrence of DCI while seven patients were untreated, but the association between low CSF Hp levels and protection from DCI was seen in both treated and untreated groups (×3 lower in all patients, ×2.8 lower in untreated patients, and ×2 lower in interleukin-1 receptor antagonist-treated patients). As hypohaptoglobinorrhachia reflects enhanced intrathecal uptake of Hp–Hb complexes, this mechanism underlies the protective effect of the Hp1-1 phenotype from vasospasm.

## Discussion

Low-grade intrathecal hemolysis may occur after mild traumatic brain injury, atherosclerosis, infections or neuroimmunological disorders, especially if perivascular inflammation is a feature. In severe intracranial hemolysis, as occurs in SAH, the intrathecal Hb-scavenging system is clearly overwhelmed. Unlike severe intravascular hemolysis, when hemoglobinemia is always accompanied by an absolute ahaptoglobinemia, free extracellular Hb occurs in the presence of Hp within the intrathecal compartment in SAH. This indicates saturation of Hb-scavenging systems within the CNS. Hence augmentation of surface CD163 expression by pharmacological inhibition of the enzyme involved in sCD163 shedding, ADAM17 (Hintz *et al.* 2002; Etzerodt *et al.* 2010), is a potential therapeutic avenue in SAH.

The majority of Hb was not bound to Hp in this study. This leaves a large amount of Hb within the CSF to drain freely out of the CNS across the BCB down a steep concentration gradient, an underestimated route of clearance from the CNS. The systemic CD163-Hp–Hb system is several log-fold more capacious than its intrathecal counterpart, and is appropriately placed to come to the rescue of the CNS after SAH. This is not the first time that the CNS has been observed to rely on systemic resources in view of its specialization; notable other instances include adaptive immunity and gluconeogenesis. It is difficult to estimate the relative contribution of intrathecal and systemic Hb-scavenging in SAH. However, the noticeable effect on systemic Hp levels, despite the dilution effect that accompanies brain-to-blood diffusion, indicates a major role for Hb efflux and systemic Hb-scavenging. Hb efflux should come as no surprise because Hb possesses favourable biochemical properties for diffusion across the BCB: size of ∼50 Å (Pertuz *et al.* 1960), *p*I of 7.2 (Mazzeo and Krull 1991) and good solubility.

The therapeutic implications of enhancing Hb clearance are being increasingly recognised. For patients with significant blood load, mechanical drainage of subarachnoid and intraventricular blood using a ventriculostomy or lumbar drain is likely to be the most effective way of clearing Hb (Klimo *et al.* 2004; Al-Tamimi *et al.* 2011; Demetriades and Tolias 2011). This approach has the potential additional advantage of lowering the intracranial pressure and thereby enhancing cerebral blood flow. However, the use of lumbar drains introduces further risks to the critically ill patient, such as epidural hematoma, neurological deficit and meningitis. Another potential means of enhancing the Hb clearance is through the modulation of BCB permeability. Hyperosmolar agents have been demonstrated to increase the permeability of the BCB (Rapoport 2000) and could therefore facilitate Hb efflux. Such agents may include hypertonic saline or mannitol. The latter may be contraindicated in patients with incipient DCI given the diuretic effect and potential decrease in cerebral blood flow and in patients with hypernatremia. The use of hypertonic saline is likely to be much better tolerated by this cohort of patients given that it actually increases cerebral blood flow and improves blood rheology (Tseng *et al.* 2003).

The Hp1-1 phenotype has a protective effect on susceptibility to vasospasm as shown by clinical and experimental studies (Borsody *et al.* 2006; Chaichana *et al.* 2007); this has been confirmed in this study. Furthermore, the underlying mechanism is demonstrated. Despite differences in Stokes-Adam radius and permeability across the BCB between Hp forms, the effective intrathecal THBC does not differ between phenotypes. Instead, evidence was found supporting enhanced Hp1-1 uptake compared with Hp2, consistent with *in vitro* evidence (Asleh *et al.* 2003). A study of intracerebral hemorrhage in mice showed that transgenic knockout and over-expression of Hp resulted in worse and better outcomes respectively (Zhao *et al.* 2009). In the present study, hypohaptoglobinorrhachia imparted a better prognosis, but this was because of enhanced Hb clearance. Hence, from a therapeutic perspective, supplementation of Hp1-1 would appear to be a potential strategy.

Further study of intrathecal Hb metabolism in SAH is required. In particular, the hemopexin-CD91 system is likely to play an important downstream role by scavenging heme, a breakdown product of extracellular Hb. Both hemopexin and CD91 are predominantly expressed by neurones in the CNS (Moestrup *et al.* 1992; Li *et al.* 2009). However, augmentation of the hemopexin-CD91 system may have its own hazards because neuronal iron overload results in neurotoxicity (Bishop and Robinson 2001). However, therapeutic manoeuvres aimed at shifting Hb out of the CNS, without affecting neuronal iron load, would appear to be more promising.

## Abbreviations

ADAM: a disintegrin and metalloproteinase
BCB: blood–CNS barrier
CT: computerized tomography
DCI: delayed cerebral ischemia
Hb: hemoglobin
Hp: haptoglobin
SAH: subarachnoid hemorrhage
SDS: sodium dodecyl sulphate
TBS-T: Tris-buffered saline containing 0.5% Tween-20
THBC: total hemoglobin-binding capacity

## Supporting information

**Appendix S1.** Supporting information.

As a service to our authors and readers, this journal provides supporting information supplied by the authors. Such materials are peer-reviewed and may be re-organized for online delivery, but are not copy-edited or typeset. Technical support issues arising from supporting information (other than missing files) should be addressed to the authors.
