# Supplementary material for: The intrathecal CD163-haptoglobin–hemoglobin scavenging system in subarachnoid hemorrhage
Source: J Neurochem. 2012 Jun;121(5):785–92. doi: 10.1111/j.1471-4159.2012.07716.x (PMC3412209; doi:10.1111/j.1471-4159.2012.07716.x)
Supplement: Supplementary file 1 [file jnc0121-0785-SD1.doc]

**Supporting Information**

**Appendix S1**

Calculation of THBC

The THBC was developed as a concept to quantitate the capacity of Hp to bind Hb. Because the THBC is expressed as the absolute mass of Hb that can be bound by Hp, this is independent of Hp phenotype. An example is set down below:

- 1 mole of Hp monomer binds 1 mole of Hb dimer (free Hb tetramer rapidly & spontaneously dissociates into dimer)
- The average molecular weight of Hp monomer is 53 (if the molecular weights of the α1 and α2 chains are averaged) while the molecular weight of Hb dimer is 34
- Therefore 53ng of Hp monomer will bind 34ng of Hb dimer
- Therefore if CSF total Hp = (834x150)ng, the THBC of CSF is (834x150X34)/53 = ~100μg Hb dimer

Please note that calculations are approximate.

Calculation of quotients

All quotients (ie QsCD163 and Qalb) were derived by simply dividing the CSF concentration by the serum concentration.

Calculation of intrathecal synthesis

The % of intrathecally produced sCD163 was calculated as follows, according to Reiber 2010:

- The CSF concentration of sCD163 derived from the circulation was calculated based on the CSF/serum albumin quotient: serum sCD163 x (CSF/serum albumin quotient)
- The % sCD163 produced intrathecally was calculated by subtracting the above value from the absolute CSF concentration, and expressing the result as a percentage of the absolute CSF concentration

Calculation of Hb-binding capacity

The Hb-binding capacity was calculated as follows:

- 1 mole of Hp monomer binds 1 mole of Hb dimer (free Hb tetramer rapidly & spontaneously dissociates into dimer)
- The average molecular weight of Hp monomer is 53 (if the molecular weights of the α1 and α2 chains are averaged) while the molecular weight of Hb dimer is 34
- Therefore 53ng of Hp binds 34ng of Hb or 10^-12^ moles of Hb
- Therefore 799ng/ml Hp will bind 15x10^-12^ mol/ml of Hb

Calculation of sCD163 index

The sCD163 index was calculated by dividing QsCD163 by Qalb, as in Reiber 2010. The sCD163 index is therefore unit-less.
